# High resolution coronary MR angiography at 7 Tesla: comparison with standard bright blood and black blood imaging

**DOI:** 10.1186/1532-429X-16-S1-M5

**Published:** 2014-01-16

**Authors:** Maurice Bizino, Cosimo Bonetti, Rob J van der Geest, Pieter J van den Boogaard, Maarten J Versluis, Andrew G Webb, Hildo J Lamb

**Affiliations:** 1 Department of Radiology, Leiden University Medical Center, Leiden, Netherlands

## Background

The aim for ultrahigh field coronary MR angiography (MRA) is to convert the benefit of increased signal-to-noise ratio (SNR) to improved spatial resolution and thereby better vessel edge sharpness (VES), while vessel conspicuity is preserved. Furthermore, black blood coronary MRA has been shown to be an alternative technique to detect arterial remodeling in patients with coronary artery disease, but has never been performed at 7 Tesla. Therefore, the purpose of this study was to compare high resolution (HR) coronary magnetic resonance angiography (MRA) to standard bright blood (BriB) and black blood (BB) imaging at 7 Tesla.

## Methods

Twenty-two healthy volunteers underwent navigator-gated 3D imaging of the right coronary artery (RCA) at 7 Tesla using three sequences: BriB (reference scan), HR and BB. Image post processing involved newly developed multiplanar reformatting to straighten the RCA. Image quality was determined by VES, SNR, contrast-to-noise ratio (CNR), visible vessel length and vessel diameter.

## Results

VES was statistically significantly higher in HR as compared to BriB (0.57 ± 0.1 vs 0.46 ± 0.06; p < 0.001), BriB was not significantly different from BB (0.44 ± 0.12; p = 0.858). SNR was higher in BriB than HR (115.6 ± 49.8 vs 33.4 ± 11.4; p < 0.001) and BB (38.3 ± 10.7; p < 0.001). CNR was highest in BriB (52.2 ± 26.7), as compared to HR (18.3 ± 7.6;p < 0.001) or BB (13.1 ± 4.9; p = 0.009). Visible vessel length and vessel diameter were similar for BriB, HR and BB (P > 0.05).

## Conclusions

High resolution coronary MRA at 7 Tesla improves vessel edge sharpness as compared to standard bright blood imaging. Black blood coronary MRA at 7 Tesla is feasible, whereas image quality is lower as compared to standard bright blood imaging.

## Funding

Funding: This research was performed within the framework of the Center for Translational Molecular Medicine (CTMM;http://www.ctmm.nlhttps://mail.lumc.nl/owa/redir.aspx?C=wk8R_jbyx0eYLbnBRS34KvWsBM9 MVNAIuYK7UjDv9cbWi1XnFTmyBKvLOW9VqUWZjR0dP2GiTSk.&URL=http%3a%2f%2fwww.ctmm.nl), project PREDICCt (grant 01C-104).

**Figure 1:**
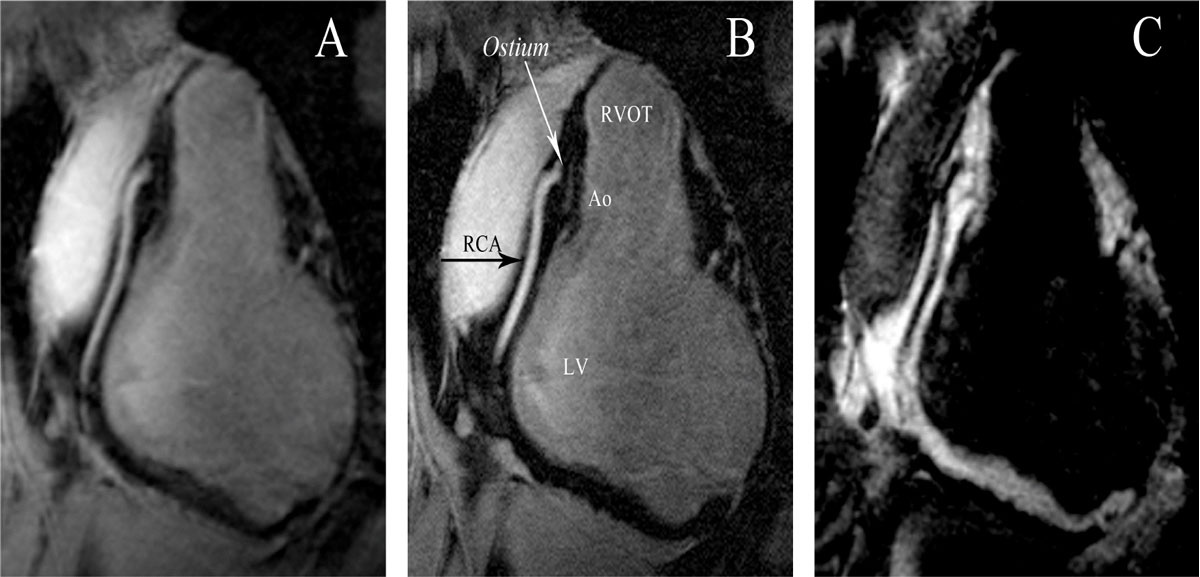
**Magnetic resonance angiograms of the right coronary artery (RCA) in a single healthy volunteer obtained with three different sequences**. A: The bright blood image shows good fat suppression and vessel conspicuity. B: High resolution sequence produces an image with clearly defined vessel borders, the contrast between blood and epicardial fat enables good identification of the RCA. Several structures can be identified in this image: the ostium and a portion of the RCA. RVOT = right ventricular outflow tract, Ao = aortic root, LV = left ventricle. C: Image acquired with black blood sequence shows a less clearly defined vessel border as compared to panel A and B. Note the suboptimal blood suppression proximally and in the distal segment.

**Figure 2:**
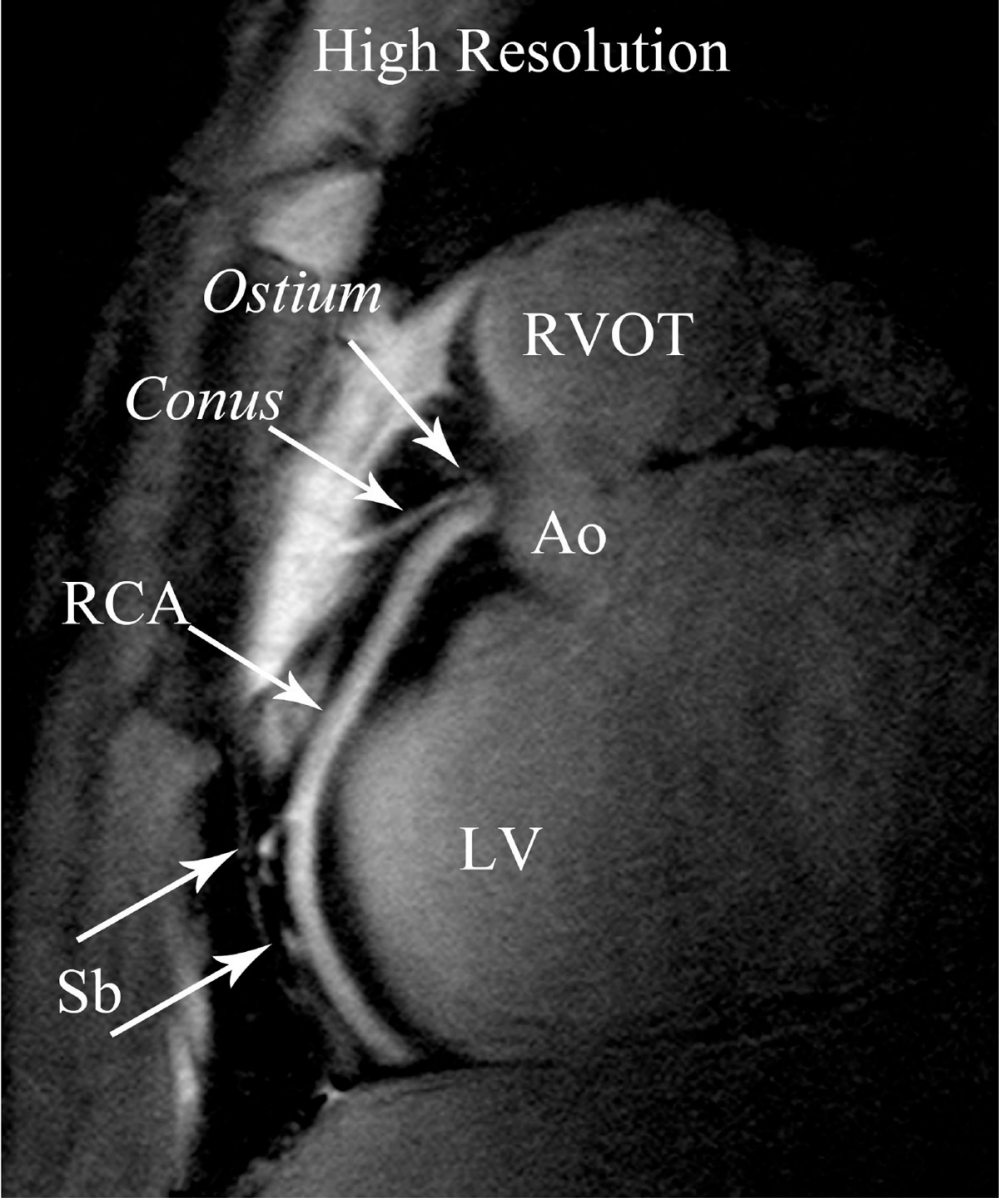
**High resolution double oblique volume targeted plane parallel to the right coronary artery (RCA)**. Note the well-defined borders of the RCA and fat suppression. In this figure it is possible to identify the ostium of the RCA, the conus, side branches (Sb). aortic root (Ao), left ventricle (LV) and right ventricular outflow tract (RVOT).

